# The role of distinct BRD4 isoforms and their contribution to high-grade serous ovarian carcinoma pathogenesis

**DOI:** 10.1186/s12943-021-01424-5

**Published:** 2021-11-10

**Authors:** Ana Luiza Drumond-Bock, Magdalena Bieniasz

**Affiliations:** Aging and Metabolism Research Program, Oklahoma Medical Research Foundation, Oklahoma City, OK 73104 USA

**Keywords:** BRD4, High-grade serous ovarian carcinoma, Gene amplification, BET inhibitors

## Abstract

High-grade serous ovarian carcinoma (HGSOC) is the most aggressive type of ovarian cancer, often diagnosed at advanced stages. Molecularly, HGSOC shows high degree of genomic instability associated with large number of genetic alterations. *BRD4* is the 4th most amplified gene in HGSOC, which correlates with poor patients’ prognosis. *BRD4* is constitutively expressed and generates two proteins, BRD4 long (BRD4-L) and BRD4 short (BRD4-S). Both isoforms contain bromodomains that bind to lysine-acetylated histones. Amongst other functions, BRD4 participates in chromatin organization, acetylation of histones, transcriptional control and DNA damage repair. In cancer patients with amplified *BRD4*, the increased activity of BRD4 is associated with higher expression of oncogenes, such as *MYC*, *NOTCH3* and *NRG1*. BRD4-driven oncogenes promote increased tumor cells proliferation, genetic instability, epithelial-mesenchymal transition, metastasis and chemoresistance. Ablation of BRD4 activity can be successfully achieved with bromodomain inhibitors (BETi) and degraders, and it has been applied in pre-clinical and clinical settings. Inhibition of BRD4 function has an effective anti-cancer effect, reducing tumor growth whether ablated by single agents or in combination with other drugs. When combined with standard chemotherapy, BETi are capable of sensitizing highly resistant ovarian cancer cell lines to platinum drugs. Despite the evidence that *BRD4* amplification in ovarian cancer contributes to poor patient prognosis, little is known about the specific mechanisms by which BRD4 drives tumor progression. In addition, newly emerging data revealed that BRD4 isoforms exhibit contradicting functions in cancer. Therefore, it is paramount to expand studies elucidating distinct roles of BRD4-L and BRD4-S in HGSOC, which has important implications on development of therapeutic approaches targeting BRD4.

## Background

Ovarian carcinoma remains one of the deadliest malignancies in the United States, rating amongst the top five causes of death for women between 40 and 79 years old [1]. High-grade serous ovarian carcinoma (HGSOC) is the most prevalent and most aggressive histotype of ovarian cancer. Clinical data revealed that in the majority of patients, HGSOC was detected at advanced stages, estimating that more than 75% of the cases were diagnosed at stage III and IV [2]. The advanced tumor stage and the poor survival rate of ovarian carcinoma patients [2, 3] reinforce the need for more rigorous studies to improve the knowledge surrounding HGSOC tumor initiation and progression, as well as response to treatments.

The analysis of genomic landscape of HGSOC tumors [4, 5] identified prevalent alterations of *TP53* gene, which is mutated in 96% of the patients. Another commonly observed genetic alteration is the loss of function of *BRCA1* and *BRCA2*, either by mutation or by epigenetic silencing [4, 6]. Loss of *BRCA1/2* leads to a deficient DNA repair and impaired homologous recombination (HR) [7] resulting in chromosomal instability [8]. HR is found to be defective in approximately 50% of the patients with HGSOC [4]. Approximately 13% of the *BRCA1/2* mutations can be attributed to inherited germline mutations [4, 6]. However, there is a subgroup of patients in which HGSOC-associated genomic instability occurs in a non-hereditary manner, and is often characterized by frequent genetic alterations (including genes amplification and deletion) in somatic cells [4, 9]. Data generated by The Cancer Genome Atlas [4] reported that the top three most common focal amplifications found in HGSOC patients encoded the genes *MECOM*, *CCNE1* and *MYC.* The *BRD4* gene represents the 4th most frequent somatic amplification in HGSOC [10], and its amplification is present in 18% of ovarian cancer patients [4, 10]. The majority of BRD4-amplified tumors shows no *BRCA1/2* alterations [11] suggesting that genomic instability in those tumors could be driven by mechanisms other than DNA repair deficiency.

BRD4 belongs to the Bromodomain and Extra-Terminal domain family of proteins (BET) [12, 13], which are known for their ability to bind to acetylated histones [14]. As an important transcriptional co-activator, BRD4 participates in several relevant processes in cancer, including DNA damage repair [15, 16] and cellular stress response [17, 18]. *BRD4* amplification [10, 11, 19] and/or overexpression [20] in ovarian tumors is often associated with a poor disease outcome [10, 11, 19–21]. The substantial impact of *BRD4* aberrations on patients’ prognosis makes BRD4 an excellent candidate for basic and pre-clinical research to facilitate the development of targeted therapies. In fact, several Phase I and II clinical trials have been initiated using BET inhibitors (BETi), alone or in combination with other drugs [22–27].

**Fig. 1:**
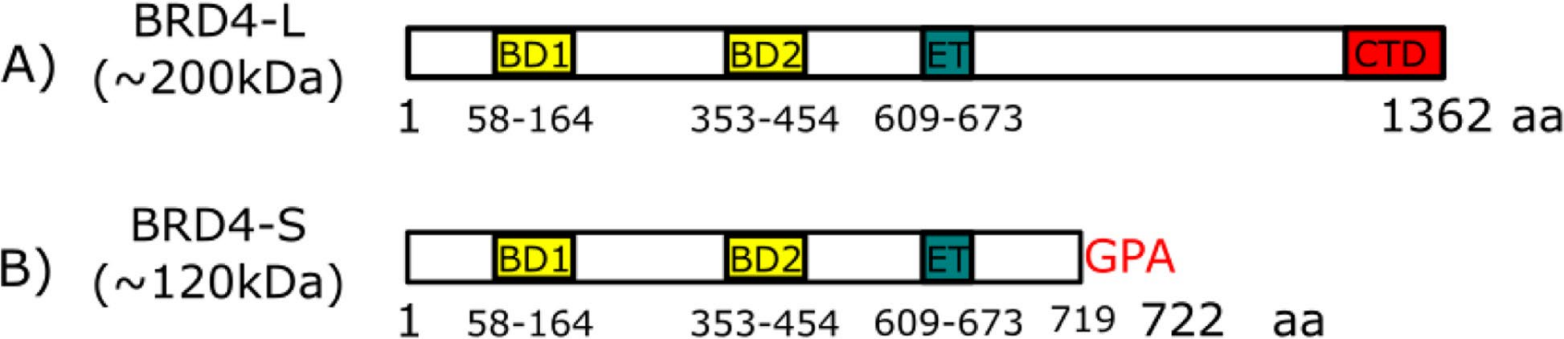
Structure of BRD4 isoforms. **a** BRD4 long isoform (BRD4-L) is a protein of approximately 200 kDa that contains two tandem bromodomains (BD1 and BD2), one extra-terminal domain (ET) and a C-terminal domain (CTD). **b** BRD4 short isoform (BRD4-S) is a protein of approximately 120 kDa that contains two tandem bromodomains (BD1 and BD2), one extra-terminal domain (ET) and a terminal domain composed of three amino acids: glycine (G), proline (P) and alanine (A). aa: amino acid

**Fig. 2:**
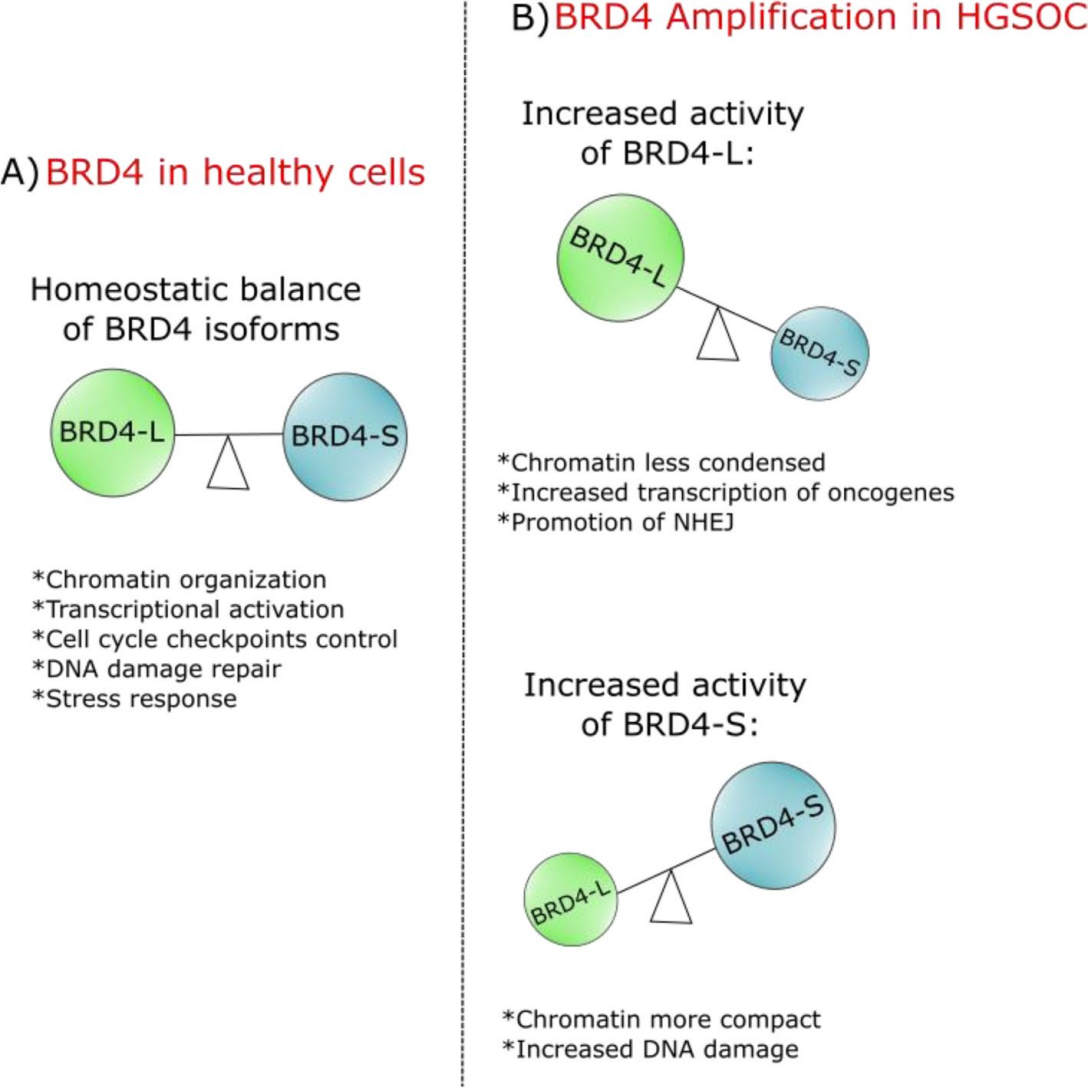
The distinct roles of BRD4 isoforms. **a** Healthy cells show balanced levels of BRD4-L and BRD4-S isoforms performing their respective homeostatic functions. **b** In patients with High-Grade Serous Ovarian Carcinoma (HGSOC), the amplification of *BRD4* results in different levels of BRD4 isoforms implicated in distinct tumor promoting functions

The *BRD4* gene encodes two main isoforms, BRD4 long (BRD4-L) and BRD4 short (BRD4-S) (Fig. 1) [10, 28, 29]. Studies indicate that a fine control of the BRD4 mRNA splicing is required to generate a balanced, constant ratio of both isoforms ensuring the homeostatic work of the protein [28] (Fig. 2). Recent data demonstrated that disruption of the balance between the two BRD4 isoforms may occur in certain diseases leading to significant biological consequences [10, 30, 31]. Further, BRD4-L and BRD4-S show different interaction patterns and distinct dynamics of transcriptional activity indicating divergent roles of these isoforms in the regulation of target genes. For instance, when overexpressed [28, 32], BRD4 isoforms tend to have opposing functions in breast cancer, BRD4-S exhibits oncogenic properties, while BRD4-L has a tumor suppressor role [30, 31]. These findings emphasize the need to depict the biological role of individual *BRD4* isoforms in respective diseases, including ovarian cancer, to facilitate development of therapeutic interventions.

Hence, the purpose of this article is to deliver a brief overview of the functions of both BRD4 isoforms in healthy and cancerous cells. We provide an insight into the potential mechanism of how BRD4 dysfunction contributes to the development and progression of HGSOC. Further, we discuss the therapeutic strategies to inhibit this oncogene, which may lead to the development of new treatment strategies for patients with ovarian cancer.

### The role of distinct BRD4 isoforms

#### Structure and function of BRD4 isoforms

**Fig. 3:**
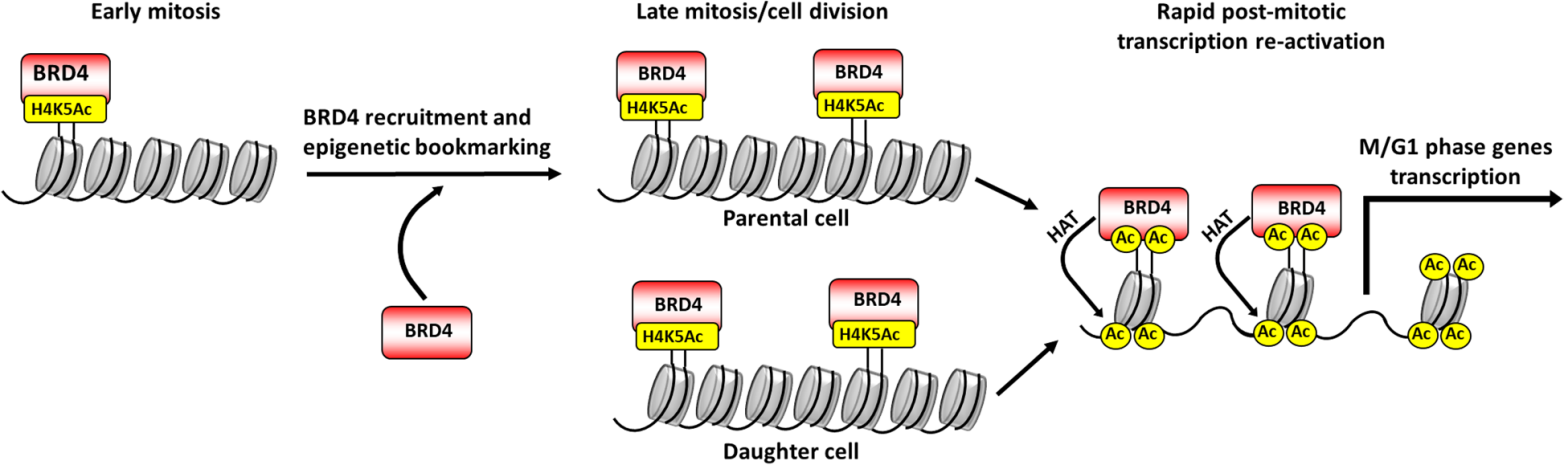
Epigenetic bookmarking. During early mitosis, the acetylation of histone H4 in lysine 5 (H4K4Ac) increases. BRD4-L is recruited to the chromatin and binds to lysine-acetylated histones (Ac). After mitotic division, BRD4-L bound to chromatin is transmitted to daughter cells and promotes rapid post-mitotic transcriptional re-activation of genes regulating M/G1 transition of the cell cycle

BRD4 isoforms belong to the BET protein family [12, 13] characterized by the presence of two tandem bromodomains (BD) and one extra terminal domain (ETD) [12] (Fig. 1). BRD4 was first described in the early 2000’s [13] as a murine protein and referred to as mitotic chromosome-associated protein (MCAP). At that time, scientists described MCAP as a protein that is distributed uniformly in the nucleus during interphase of the cell cycle, which becomes associated with condensed chromosomes throughout mitosis [33, 34]. The fact that BRD4 (then MCAP) remained associated with chromatin in the cell cycle phase in which most proteins are released into the cytoplasm has lead these authors to propose that BRD4 plays an important role in the chromatin dynamic during mitosis [13]. Further studies have defined BRD4 isoforms as epigenetic markers (Fig. 3) that bind to acetylated core histones and are transmitted to daughter cells, establishing the histone code across cell division [35–37].

Thus far, the majority of the studies investigating the function of BRD4 focused on its long isoform (Table 1). BRD4-L contains two tandem bromodomains, one extra-terminal domain, and a C-terminal domain (Fig. 1A). While BRD4 bromodomains and the extra-terminal domain are highly conserved amongst BET proteins, the C-terminal domain is highly unique conferring BRD4-L a distinct transcriptional co-activator function [38]. Although BRD4-L is more active and more frequently expressed isoform, BRD4-S appears to be highly relevant as well. Studies revealed that BRD4-S is the predominant isoform binding to modified histones [38] with a stronger binding affinity to the chromatin than BRD4-L [39]. BRD4-S has the same N-terminal segment as BRD4-L, but lacks the C-terminal domain (Fig. 1B). Additionally, BRD4-S contains three unique C-terminal residues (GPA), not present in BRD4-L [39, 40]. To date, no specific function has been attributed to the GPA residues of BRD4-S.

**Table 1:** Isoform-specific functions of BRD4

Isoform	Function	Ref.
	Serves as transcriptional co-activator	[41, 42]
BRD4 isoform long (BRD4-L)	Promotes RNA transcription via RNA Polymerase II pause-release	[41–43]
	Has intrinsic histone acetyltransferase activity	[44]
	Maintains higher-order chromatin structure	[38]
	Induces expression of genes involved in the DNA repair pathway NHEJ	[15, 16]
	Provides structural support for NHEJ protein complex	[15, 16, 45]
	Regulates transcription of primary response genes	[17]
	Regulates transcription of antioxidant genes via interaction with NRF2	[18]
	Indirectly induces generation of ROS via KEAP1-NRF2 pathway	[46]
	Has transformative potential in epithelial ovarian cells	[10]
	Predominantly binds to lysine-acetylated histones	[38]
BRD4 isoform short (BRD4-S)	Promotes chromatin compaction	[38]
	Incorporates BRD4 condensation into the chromatin	[39]
	Sustains transcription of proliferative genes in cancer cells via phase separation	[39]
	Inhibits DNA damage repair	[15, 47]
	Promotes oncogenic properties in cancer	[10, 28, 30]

*Abbreviations*: *NHEJ* non-homologous end joining; *ROS* reactive oxygen species

#### Chromatin organization and HAT function of BRD4 isoforms

The bromodomains of the BET proteins are structures capable of recognizing and binding to lysine-acetylated histones [38, 48, 49]. BRD4 isoforms bind to the chromatin when recruited and detach when not needed, working as an “on and off” switch [37]. BRD4 predominantly binds to histone H4 acetylated in lysine 5, 8 and 12 (H4K5Ac/H4K8Ac/H4K12Ac) [36, 37, 50]; BRD4 isoforms can also associate with histone H3, acetylated in lysine 14 (H3K14Ac) with a somewhat lower affinity [37]. The higher the level of histone acetylation, the stronger the bond between BRD4 proteins and the chromatin, and the less likelihood of either isoform unbinding from the acetylated lysine [37]. Experimental conditions that disrupt the binding of endogenous BRD4 isoforms to the acetylated histones result in chromatin rupture, decompaction, and fragmentation [38].

**Fig. 4:**
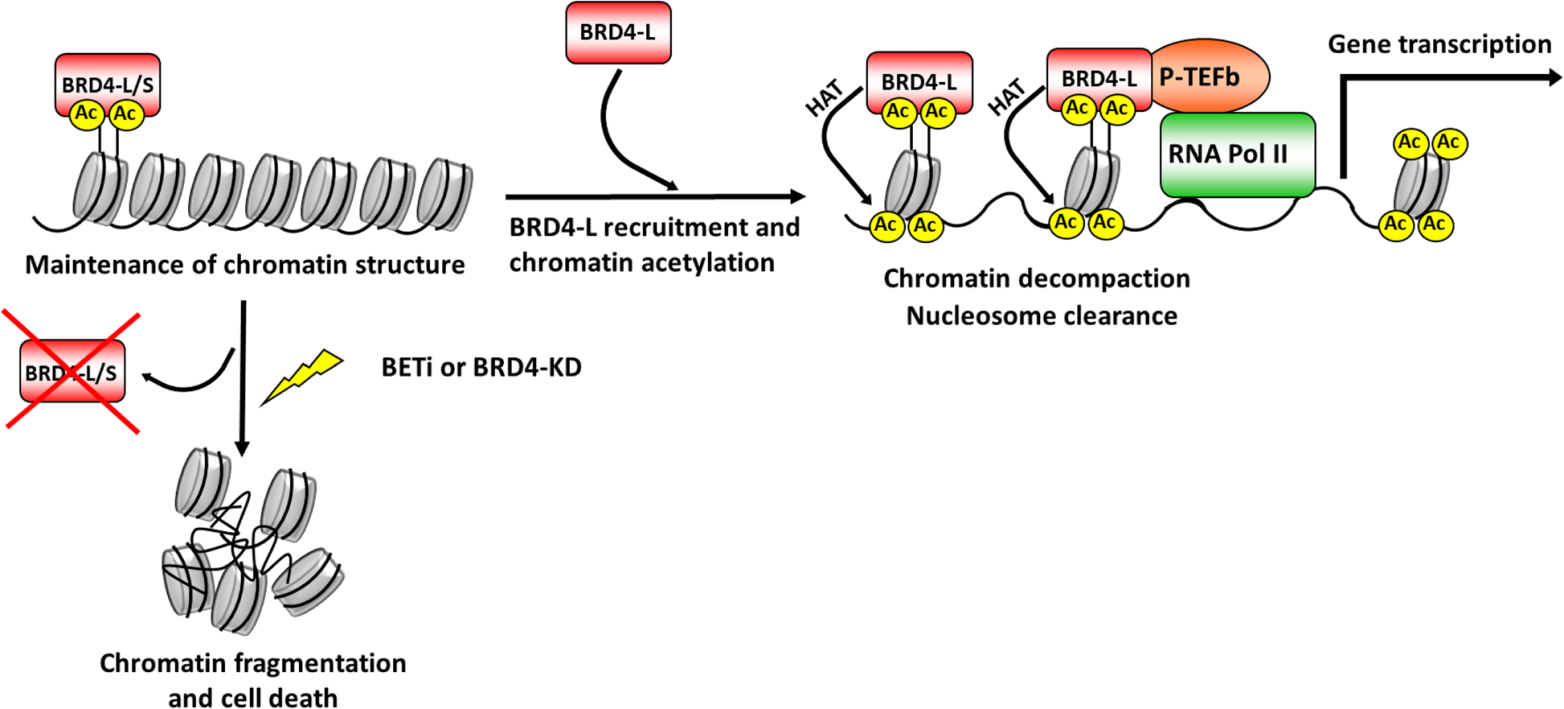
The role of BRD4 in chromatin organization and gene transcription. BRD4-L and BRD4-S bind to lysine-acetylated histones (Ac) to maintain the higher order of chromatin. Treatments that prevent binding of BRD4-L/S to chromatin induce the chromatin fragmentation. BRD4-L promotes histone acetylation (HAT) recruiting more BRD4-L molecules. BRD4-L associates with the positive transcription elongation factor b (P-TEFb). BRD4-L and P-TEFb re-activate gene transcription via pause release of the RNA polymerase II (RNA Pol II). BETi: Bromodomain inhibitors; BRD4-KD: BRD4 knockdown

Reports have found that BRD4-L bromodomains establish intermolecular interactions promoting a formation of BRD4-L-BRD4-L complexes [38], and might also be required for the establishment of nucleosome-nucleosome associations. Furthermore, BRD4-L’s C-terminal (Fig. 1A) domain appears to be involved in ensuring proper organization and maintenance of chromatin, as it controls BRD4-L biding to acetylated histones [37, 38]. Therefore, the combination of both interactions, BRD4-L-chromatin and BRD4-L-BRD4-L, appears to be essential for the higher-order chromatin organization (Fig. 4). Likewise, a synthetic protein [37] representing BRD4-S isoform (Fig. 1B) appears to be relevant for the structural support of the chromatin due to its stronger and more stable binding to histones, leading to a compact chromatin conformation [37]. Under experimental conditions, exogenous BRD4-S is capable of displacing endogenous forms of BRD4-L [38]. The conformational changes of chromatin structure caused by impairment of BRD4 binding to the histones alters basic cellular functions, such as DNA replication and gene expression [51].

Recent studies showed that BRD4 might also play an active role in the histone lysine-acetylation [44]. One of the first indicators that BRD4 might function as a histone acetyltransferase (HAT) is that drug-induced depletion of BRD4 isoforms leads to hypoacetylation of histone H3 and H4 [52]. In addition, treatments with BETi are associated with reduced levels of acetylated histones [53]. In a recent study, Devaiah et al. [44] demonstrated that BRD4-L has an intrinsic HAT activity, carrying out the acetylation of H3 and H4 lysines of nucleosomal histones (Fig. 4). In addition, BRD4-L acetylates H3K122 residues located in the globular core of the H3 histone [44], where the DNA-histone bond is the strongest [54]. Through the histone acetylation process, BRD4-L actively evicts nucleosomes from chromatin, leading to decompaction of the chromatin structure, which becomes substantially more accessible to the transcriptional machinery. Therefore, by regulating the accessibility of chromatin to transcriptional complexes, BRD4-L plays an important role in inducing gene transcription.

#### BRD4 isoforms as transcriptional co-activators

During gene transcription, RNA Polymerase II (Pol II) is recruited to gene promoter regions [55]. After transcription initiation, Poll II can promote RNA elongation or simply stall at the promoter proximal region, remaining in a paused state [55, 56]. BRD4 plays an important role in the re-activation of paused Pol II via recruitment and activation of the Positive Transcription Elongation Factor b (P-TEFb) [41, 42]. BRD4-L interacts with P-TEFb via its C-terminal domain (Fig. 1) [43], and it might bind to either or both subunits of P-TEFb known as CycT1 and Cdk9 [41, 43]. Together, BRD4-L and P-TEFb form a transcriptional activation complex (Fig. 3), which phosphorylates both serine sites of Pol II, initiating RNA elongation [57]. BRD4-L and P-TEFb interact throughout interphase, but dissociate during early mitosis [41], when the transcription process halts. These protein interactions increase dramatically in cells progressing from late mitosis to early G1 phase of the cell cycle [58]. These findings, and the presence of BRD4 isoforms associated with chromatin throughout mitosis [13], reinforce the role of BRD4-L as an epigenetic memory marker (Fig. 3), which acts by recruiting P-TEFb and quickly re-activating transcription after mitosis [58]. BRD4-L appears to have a more prominent role during post-mitotic transcriptional reactivation than during baseline interphase transcription, since the inhibition of BRD4-L does not significantly affect mRNA synthesis during interphase [36].

In addition to transcriptional activation of Pol II, BRD4 isoforms associate with the transcriptional co-factor MED1 and use phase-separation to form and compartmentalize condensates of high densities of transcriptional proteins [39] around super-enhancers (SE) [59]. SE are clusters of enhancers that regulate genes important in cell identities [60], including cancer cells [60, 61]. The loss of condensate integrity significantly affects transcription on SE sites [59] BRD4-S was found to play a larger role in incorporating BRD4 condensation in the chromatin than BRD4-L [39]. BRD4-S organizes transcription factors through phase-separation to sustain transcription in chromatin for cancer cell proliferation [39].

#### Opposite roles of BRD4 isoforms in DNA damage repair

**Fig. 5:**
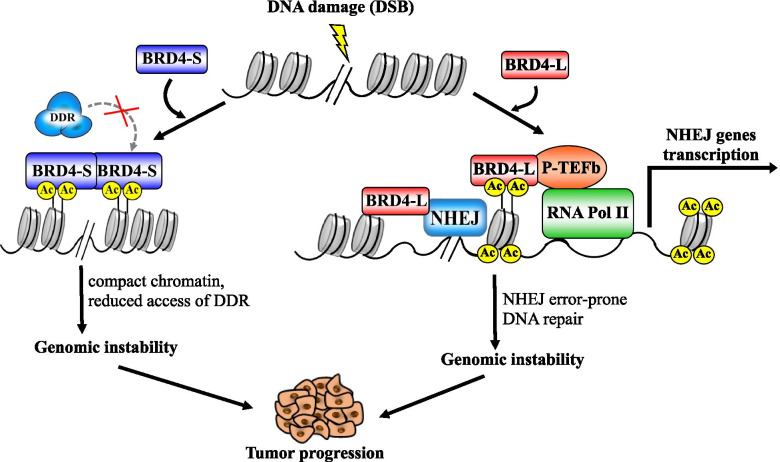
The role of BRD4 isoforms in DNA damage response. BRD4-S stably binds to lysine-acetylated histones (Ac), promoting chromatin compaction and shielding damaged DNA from the DNA damage repair (DDR) machinery. BRD4-L promotes non-homologous end joining (NHEJ) DNA repair. BRD4-L induces NHEJ genes transcription and stabilizes NHEJ protein complexes in the DNA damage site. Increased activity of either or both isoforms can lead to genomic instability due to decreased DDR and promotion of error-prone NHEJ. DSB: Double strand breaks; RNA Pol II: RNA polymerase II; P-TEFb: Positive Transcription Elongation Factor b

BRD4 is an important regulator of genes involved in DNA damage repair (DDR) 15, 16]. Li et al. [16] reported that the inhibition of BRD4 function (using BETi or shRNA) results in a decreased expression of genes involved in the DNA repair pathway known as non-homologous end joining (NHEJ) pathway. NHEJ is a mechanism of double strand breaks (DSB) repair that mediates direct re-joining of DNA strands with damaged termini [62]. In addition to transcriptional regulation of genes involved in DNA repair, BRD4-L directly binds and modulates the function of protein complexes involved in NHEJ [45]. In the presence of DSB, the chromatin undergoes modifications including H4 histone acetylation and H2AX histone phosphorylation [15], which is followed by the recruitment of BRD4-L that binds to chromatin regions with damaged DNA. Then, BRD4-L functions as an anchorage for DNA repair proteins (Fig. 5), similarly to its role in transcriptional activation. Interestingly, BRD4-S has been reported to have an opposite effect in the DDR [15, 47], acting as an endogenous inhibitor of DNA repair complexes. BRD4-S binds stably to DNA molecules [37] and in the presence of DNA damage, it shields the chromatin from the DDR machinery [15, 47, 63]. In addition, BRD4-S recruits components of the condensing II complex inducing chromatin condensation, which impedes propagation of the DDR [47].

### BRD4 contribution to high-grade serous ovarian carcinoma pathogenesis

#### Amplification of BRD4 in HGSOC patients

Approximately 18% of the patients with high-grade serous ovarian carcinoma present somatic amplification of *BRD4* [4, 10], which is often associated with increased *BRD4* gene and protein expression [10, 11, 64]. HGSOC patients harboring focal amplification of the chromosome 19, in regions that encompass *BRD4* (19p13.2) [13, 19, 65], tended to show advanced-stage ovarian cancer (97% of the patients were diagnosed with stage III or IV tumors) [19]. These patients also presented considerable worse ovarian carcinoma prognosis. In consistency with this report, other studies have correlated BRD4-amplification with worse patient survival [11, 20, 21, 64, 66]. Amongst chemotherapy resistant patients [19], the majority of patients (61%) had *BRD4*-amplified tumors. As we will discuss further, BRD4 appears to be directly related to chemoresistance via regulation of ALDH activity [67] and promotion of DNA damage repair [68].

In HGSOC patients, the amplification of *BRD4* is often associated with the amplification of other genes [11, 19, 21]. For instance the cyclin E 1 gene (*CCNE1*) [11] is amplified in 46–48% of patients with *BRD4* amplification [20, 21]. Patients with co-amplification of both genes demonstrated worse survival than patients with only one of the genes amplified. Just as with *BRD4*, amplification of *CCNE1* in these patients is accompanied by increased gene expression and protein levels of CCNE1 [11, 21]. *CCNE1*-amplified tumors are characterized by aberrant DNA replication, DNA replication stress and high levels of genomic instability [21]. Researchers suggest that the aberrant activation of cyclin pathway contribute to the genomic instability in patients harboring *BRD4* amplification [13, 19, 65]. Finally, it is important to point out that *BRD4* amplification and *BRCA1/2* mutations tend to be mutually exclusive in patients with HGSOC [11, 19], which suggests that the genomic instability in *BRD4*-amplified tumors is not due to the loss of BRCA functions.

#### Isoform-specific functions of BRD4 in cancer

A number of studies have correlated the amplification of *BRD4* with HGSOC progression [10, 64] and poor patient outcome [11, 20, 21, 64, 66]. Although there have been attempts to characterize the oncogenic effect of *BRD4* amplification in HGSOC [10], the exact mechanism by which BRD4 elicits ovarian tumor promotion is still a matter of scientific debate. Perhaps, better understanding of the effects of *BRD4* amplification on the initiation and progression of ovarian carcinoma relies on exploring the independent functions of BRD4 isoforms. Analysis of publically available TCGA data [4] revealed that both BRD4-L and BRD4-S are overexpressed in varying ratios in HGSOC patients whose tumors harbor *BRD4* amplification [10]. Furthermore, Rhyasen et al. [10] reported that exogenous overexpression of both BRD4 isoforms in non-transformed ovarian epithelial cells showed robust colony formation in vitro, however, the short isoform of BRD4 showed a stronger tumorigenic potential than the long form of BRD4.

Emerging findings shed light on the importance of BRD4 isoforms in triple negative breast cancer (TNBC) [28, 29, 32], which is molecularly similar to HGSOC, presenting widespread genomic instability and a high mutation rate of *TP53* [4, 69]. A thorough study [28] performed in patient xenografts, cell lines and mouse models showed that, similar to what was observed in HGSOC [10], the ectopic expression of BRD4-S in TNBC tumors promotes oncogenic phenotype. Cells with BRD4-S showed increased cell proliferation, tumor growth and metastasis, while overexpression of BRD4-L suppressed these phenotypes. In addition, loss of BRD4-S function reduced cell proliferation, tumor growth and metastasis. Finally, the analysis of the percentage of individual BRD4 isoforms showed that TNBC patients with a higher BRD4-L to BRD4-S ratio presented a better overall survival than those with the lower ratio. The authors [28] concluded that BRD4 isoforms have opposing functions in TNBC and that post-transcriptional regulation may play a critical role influencing BRD4 protein isoform abundance during cancer progression.

Mechanistically, BRD4-S can exert different functions that depend on whether it associates to its target gene promoters or enhancers, and whether it interacts with BRD4-L [28]. In TNBC, in the absence of BRD4-L, BRD4-S interacts with homeobox transcription factors and binds to enhancer regions promoting transcription of genes that modulate components of the extracellular matrix (ECM). This leads to changes of the tumor microenvironment favoring disease progression. In the presence of BRD4-L, however, BRD4-S interacted with BRD4-L and acted as co-repressor, halting the expression of tumor promoting ECM genes and consequently suppressing tumor development.

In summary, it appears that the functions of BRD4-L and BRD4-S are substantially affected by the expression levels of each isoform and, potentially, by their mRNA splicing ratio [28]. In fact, some studies demonstrated that different genes have different requirements of BRD4-S and BRD4-L for transcriptional activation [39]. It is possible that, just as with TNBC, the amplification of *BRD4* in HGSOC may lead to a shift in the splicing ratio of the two BRD4 isoforms resulting in BRD4-S overactivation that drives ovarian cancer pathogenesis and contribute to patients’ poor prognosis (Fig. 2). However, further studies are needed to validate this hypothesis.

#### BRD4-mediated transcriptional regulation of ovarian carcinoma oncogenes

In ovarian cancer patients, the increased activity of *BRD4* is associated with the higher expression of a variety of oncogenes [10, 20, 64, 66], many of which are positively regulated by BRD4 on transcriptional level [10, 20, 64]. *MYC* is one of the most studied oncogenes in HGSOC, which has been often associated with BRD4 [10, 20, 70], and whose expression in cancer can be down regulated by BETi [10, 71]. Chromatin immunoprecipitation experiments showed that BRD4 binds directly to *MYC* transcription start site, as well as enhancer regions in ovarian cancer cells [10]. Furthermore, it has been demonstrated that the amplification of *BRD4* correlates with overexpression of MYC in HGSOC tumors from patients [10, 20] and in vitro transformed ovarian surface epithelium [10]. In addition, a treatment of ovarian tumors with BETi suppresses MYC activity, by impairing *MYC* gene expression [10, 72, 73].

*NOTCH3* is another oncogene of high relevance in ovarian carcinoma [74–77], which has been recently identified as being directly regulated by BRD4 in HGSOC [64]. NOTCH3 pathway activation in HGSOC correlates with ovarian cancer progression [75, 76] and contributes to epithelial-mesenchymal transition (EMT) and chemoresistance [74, 77]. BRD4 binds to the promoter regions of *NOTCH3* in ovarian carcinoma [64], and inhibition of BRD4 activity is associated with lower expression of NOTCH3 mRNA and protein, as well as the expression NOTCH3 target genes.

Other studies revealed that induced expression of BRD4 isoforms in ovarian epithelial cells correlates with increased expression of *NRG1* [10], which has been shown to participate in ovarian cancer cells proliferation and metastasis [78, 79]. The *NRG1* gene encodes the protein Neuregulin 1, which is a glycoprotein with an epidermal growth factor-like domain that carries extracellular signals inside the cell by activating ErbB receptor tyrosine kinases. BRD4 directly binds to two independent *NRG1* promoter and enhancer sites [10], and NRG1 has been identified as a direct effector of BRD4 in ovarian carcinoma. Recent reports have found *NRG1* to be a key gene associated with chemotherapy response in ovarian carcinoma and its downregulation correlated with tumor sensitivity to treatment [80]. Hence, transcriptional modulation of the NRG1 pathway might be one of the mechanisms through which BRD4 plays a role in ovarian cancer patients’ response to chemotherapy, however, further studies are required to validate such mechanism.

#### Modulation of oxidative stress response in HGSOC

Oxidative stress is considered a secondary hallmark of cancer [8] that plays an important role in ovarian carcinoma pathogenesis [81], often being associated with a development of chemoresistance in cancer cells [82, 83]. BRD4 is involved in the mechanism of cellular response to oxidative stress, via regulation of the KEAP1/NRF2 signaling network [18, 46], which is one of the major oxidative stress response pathways [84]. Under baseline conditions, BRD4 constitutively binds to promoter sites of genes regulated by the transcription factor NRF2 [18, 46], and plays a role as both a gene expression inhibitor as well as activator, a mechanism that is cell-type dependent [18]. One example of genes regulated by BRD4-NRF2 axis is the heme oxygenase 1 (*HMOX1*) encoding an inducible enzyme HMOX1 that actively eliminates endogenous ROS [85]. *HMOX1* is considered to be an oncogene in ovarian cancer [81] and its high expression promotes proliferation in ovarian carcinoma cells [86]. In addition, HMOX1 overexpression has been found to be a predictor of worse prognosis in ovarian carcinoma patients [86]. Studies also showed that BRD4 can directly bind to *HMOX1* promoter region, in an KEAP1/NRF2-independent manner stimulating *HMOX1* gene expression, which then participates in the maintenance of endogenous ROS levels [18, 46].

#### Promotion of genomic instability and chemoresistance in HGSOC

Impaired DNA repair mechanisms can lead to genomic instability [87]. This is particularly the case in HGSOC, which is characterized by high genomic instability [4, 62]. Recent studies indicate that genomically instable cancer phenotype in patients with BRD4 amplification could be attributed to less efficient DNA repair mechanisms. For instance, in DNA damage conditions, BRD4 mediates error-prone DNA repair via NHEJ pathway [16, 45, 62] rather than error-free HR pathway. NHEJ is defective in more than 40% of ovarian carcinoma patient, which is independent of HR function [88]. In addition, the short isoform of BRD4 (BRD4-S) effectively blocks DNA repair by preventing recruitment of DDR complexes to chromatin with damaged DNA, thereby diminishing DNA repair deficiency [15, 47, 63].

Furthermore, BRD4 role in DDR might also correlate with the development of resistance in BRD4-amplified patients to DNA-damage inducing chemotherapy [19]. One of the most studied resistance mechanisms of platinum-based chemotherapy is increased DNA repair [89]. The importance of DNA repair in HGSOC is evidenced by the mechanism of action of the most effective chemotherapies inducing DNA damage, and the high incidence of DNA repair dysfunction in HGSOC [89]. BRD4 regulates single strand breaks repair by activating the protein kinase CHK1 [68], which triggers checkpoint signals to promote delay of cell cycle progression and restoration of stalled replication forks [90, 91]. Ovarian cancer cells treated with BETi show a time-dependent reduction in the levels of pCHK1, suggesting that BRD4 can regulate CHK1 signaling in response to DNA replication stress [68].

Another mechanism through which amplification of BRD4 in HGSOC patients might lead to chemoresistance is modulation of aldehyde dehydrogenase (ALDH) activity, which is regulated on transcriptional level. Reduced expression of aldehyde dehydrogenase gene *ALDH1A1* is associated with stemness properties as well as platinum resistance of ovarian cancer cells [91]. Cells with decreased expression of ALDH1A1 presented spontaneous DNA damage, in addition to dephosphorylation and consequent de-activation of CHK1, which contributed to chemosensitization of ovarian cancer cells [92]. Furthermore, ovarian cancer cells with higher activity of ALDH showed enhanced DNA repair, suggesting an important role for this protein in resistance to drug-induced DNA damage [92, 93]. BRD4 has been shown to regulate ALDH1A1 expression at a transcriptional level [67]. Increased expression of BRD4 in ovarian cancer cells resulted in an increase of ALDH activity [94], while diminished BRD4 activity decreased ALDH activity by direct suppression of ALDH1A1 gene expression [67].

#### Induction of epithelial-mesenchymal transition

Recent reports demonstrated that BRD4 is directly involved in the metastatic process in HGSOC patients [95]. The downregulation of BRD4 activity markedly reduced the invasive properties of ovarian cancer cell lines, while BRD4 upregulation augmented cell migration and invasion. The development of ovarian cancer metastasis involves several steps that often relay on the process of epithelial-mesenchymal transition [96, 97]. One of the early events involved in EMT is the loss of E-cadherin due to direct transcription inhibition [98–100] by transcriptional repressors such as Sip1/ZEB2, Snail, and Slug [97, 101]. Although there are no studies that specifically elucidate the involvement of BRD4 in the regulation of Snail1 and Slug in ovarian carcinoma, the mechanism by which these genes promote EMT is relatively conserved amongst different types of tumors [100]. A lung cancer study demonstrated that BRD4 is involved in the promotion of EMT [102], via regulation of these transcriptional repressors. The histone acetyltransferase PCAF promotes acetylation of the transcription factor ISX which interacts with the bromodomains of BRD4, forming a transcriptional activating complex. The PCAF-ISX-BRD4 complex binds to the promoter regions of Snail1 and Slug inducing their transcription [102] and promoting EMT characteristics of the tumor cells. It is plausible to speculate that a similar mechanism is at play in HGSOC patients.

### BRD4 as a therapeutic target in ovarian carcinoma

To ablate the activity of bromodomain proteins in ovarian carcinoma, particularly BRD4, a variety of small-molecule BETi have been developed and tested [10, 64, 67, 103–106]. The JQ1 compound was shown to bind to the bromodomains of BRD4 (Fig. 1), with higher affinity and stability than to other members of the BET family [107] and it has been largely used in pre-clinical studies testing BETi in ovarian cancer [67, 68, 72, 104–106, 108, 109]. JQ1 has been shown to suppress the expression of several cancer related genes in ovarian carcinoma [67, 72, 105, 110]. Although JQ1 has been reported to be a cytostatic drug suppressing ovarian cancer cell proliferation [72, 104, 106, 109, 110], some studies suggest that it also induces apoptosis, especially when used in combination with other drugs [64, 67, 72, 105, 109].

I-BET151 is another small-molecule inhibitor commonly tested in ovarian carcinoma [111–113]. I-BET151 was reported to impair tumor growth by displacing BRD4 from the chromatin, which reduced the expression of the Forkhead box protein M1 (FoxM1) transcription factor [112, 113]. FoxM1 induces the expression of genes involved in cell proliferation, therefore down-regulation of FoxM1 inhibits tumor growth [112, 113]. In addition, I-BET151 inhibits migration and invasion of ovarian cancer cells by downregulation of EMT master regulators, which suppresses the expression of matrix metalloproteinases (MMPs) such as MMP2 and MMP9 [111, 112]. MMPs are enzymes that degrade and modify the ECM mediating cell motility, invasion, and EMT phenotype [114]. Finally, the use of I-BET151 in combination with cisplatin was shown to augment the inhibitory effect of cisplatin in ovarian cancer cell lines, considerably reducing cell proliferation [112].

Combination therapy with BETi can be also very effective in overcoming chemoresistance in pre-clinical settings [72, 112, 115, 116]. JQ1, for example, effectively increases sensitivity to cisplatin of platinum-resistant ovarian cancer cell line in vitro [72]. Combination of JQ1 or BET151 with cisplatin significantly reduced cell proliferation, even in highly chemo-resistant ovarian cancer cell lines [72, 112] and primary tumor cells [72]. However, the successful use of combination therapy using BETi in ovarian cancer goes beyond sensitization of tumors to platinum drugs. Pre-clinical studies demonstrated that the pharmaceutical inhibition of BRD4 or *BRD4* knockdown contribute to homologous recombination defects (HRD), sensitizing ovarian carcinoma cells to Poly (ADP-ribose) polymerase inhibitors (PARPi), regardless of their BRCA mutation status [117, 118]. When treated with JQ1, OVCAR3 cell line demonstrated a significant increase in the expression of DNA damage repair genes and activation of the cell cycle checkpoint genes [118]. Administration of PARPi olaparib to JQ1 treated cells induced high levels of apoptosis. The cell death induced by JQ1-olaparib combination therapy was more profound than after the treatment with each drug alone indicating a synergistic antitumor efficacy. In addition, co-treatment of cancer cells with BETi and PARPi reversed the PARP-resistance in both in vitro and in vivo studies [117].

BETi have been also frequently combined with tyrosine kinase inhibitors (TKi), such as ponatinib and lapatinib [10, 106]. Tyrosine kinases are key regulators of mitogenic signaling pathways frequently implicated in oncogenesis [119]. Treatment with TKi inhibit tumor growth by suppressing essential cellular processes such as proliferation, survival and invasion. Ovarian tumor models exposed to combination therapies such as ponatinib (TKi) and dBET1 (BRD4 degrader) [106], or lapatnib (TKi) and AZD5153 (BETi) [10] induced more robust cell apoptosis and tumor regression than each drug alone. Finally, the combination of BETi with MEK inhibitors (MEKi) has been shown to efficiently and synergistically suppress ovarian carcinoma growth by inducing cell death both in vitro and in vivo [109, 120]. MEKi ablates the activity of the RAS/MAPK signaling pathway involved in cell growth and tumorigenesis [121], which has been found to be significantly increased in HGSOC [122].

Despite the extensive use of BETi in pre-clinical studies including ovarian cancer, the knowledge around the applicability of these compounds in the actual clinical set up is very limited. Currently, there are 9 reportedly active clinical trials studying BETi as anticancer drugs [27] and two of those studies rely on the participation of ovarian carcinoma patients [25, 26]. It appears that amongst the challenges associated with the use of BETi in patients, considerable side effects, such as thrombocytopenia, asthenia, fatigue, as well as digestive discomfort (nausea, anorexia, diarrhea, and vomiting) have been commonly reported by clinical studies using BETi in humans [23, 123–127]. Nonetheless, because pre-clinical data in ovarian carcinoma are so encouraging [22], especially when used in combination therapy, attempts to successfully implement BETi treatments continue to be made [127]. Table 2 summarizes the most recent information on clinical studies developed with participation of ovarian cancer patients.

**Table 2:** Clinical studies involving patients with ovarian cancer

BET inhibitor	Malignancy eligibility	Response	Ref.
INCB054329	HGSOC (solid tumors)	PD as best response	[23]
INCB057643	*BRCA*^*WT*^ HGSOC (solid tumors)	PD as best response	[23]
ODM-207	HGSOC	No result reported for HGSOC	[24]
RO6870810	Advanced OC	No result reported	[25]
BMS-986158	*BRCA*^*WT*^ OC (advanced tumors)	Under development, no results yet	[26]
ZEN-3694	Platinum-resistant OC and refractory OC	Not yet recruiting	[27]

*Abbreviations*: *HGSOC* high-grade serous ovarian carcinoma; *WT* wild type; *OC* ovarian carcinoma; *PD* progressive disease

### BETi limitations

Despite the large number of studies proposing BETi compounds as successful alternative treatment for ovarian carcinoma and other types of cancer [22], BETi have limitations. One caveat to BETi therapy is that ovarian carcinoma cells may acquire resistance following sustained treatment with BETi [110, 128–131]. JQ1 resistance, for example, can be achieved through remodeling of epigenetic markers and reactivation of the transcription of key BRD4 target genes [128]. Some reports have found that resistance to BETi can be mediated by adaptive kinome reprogramming, via activation of compensatory pro-survival kinase networks, which overcomes BET protein inhibition [110]. However, this phenotype can be reversed with the use of drugs that block respective kinases, preventing or delaying the development of resistance and enhancing the efficacy of BETi therapy. In addition, evaluation of apoptotic and proliferative response in 2 sensitive and 2 resistant cell lines to JQ1 showed that this BET inhibitor induced pro-survival autophagy via inactivation of the Akt/mTOR pathway, elucidating another potential mechanism of resistance in ovarian cancer cells [130]. As an alternative to the use of BETi, the use of proteolysis targeting chimeras (PROTACS) has been proposed with the intention to degrade BRD4 [129, 132, 133]. PROTACS, such as dBET1 and dBET6, selectively and substoichiometrically degraded BRD4 and demonstrated to be more potent antitumor activity in ovarian carcinoma than the small molecule inhibitors [132, 133].

One of the concerns in using BETi is that these drugs also show some inhibitory activity towards other members of the BET family [107, 129]. For instance, the use of high doses of BETi leads to a pan-BET inhibition, which alters a number of signaling pathways essential for normal cell function potentiating undesired side effects [128, 129, 134]. In addition, there is a likelihood of inconsistency in reports describing BRD4 functions if the BRD4 function attribution was solely based on the use of BET inhibitors [128, 135]. Therefore, it is paramount that basic and pre-clinical studies using BETi be performed in parallel with other forms of BRD4 inhibition, such as siRNA and complemented with silencing of the other members of BET [117, 136].

## Conclusion

In summary, BRD4 is a dynamic constitutively expressed protein, which participates in a variety of homeostatic processes in healthy cells, via both its chromatin binding function and its transcriptional co-activator role. Amplification of *BRD4* is amongst the top five most common somatic amplification occurring in HGSOC, and it correlates with poor patient prognosis. However, further studies are necessary to clarify the mechanisms behind the role of BRD4 in ovarian carcinoma. Because amplification of *BRD4* is more common in older patients, with advanced stages of the disease, it is likely that BRD4 plays a more pertinent role in the progression of HGSOC than in the initiation of the disease. Studies in breast cancer have demonstrated a compelling evidence that BRD4 isoforms have distinct and even contradictive functions [28], providing an insight into mechanisms that are likely at play in other tumor types including ovarian carcinoma. Therefore, it is important to expand this area of research in HGSOC to elucidate the different functions of individual BRD4 isoforms, establishing a relationship between their splicing ratio and disease progression, as well as patients’ prognosis. Lastly, it would be informative to initiate mechanistic studies investigating the physiological role of BRD4 isoforms in healthy cells, since very little is known about BRD4 isoforms (especially BRD4-S) in the non-pathological context.

## Data Availability

Not applicable.

## Abbreviations

BET: Bromodomain and extra-terminal domain family of proteins
BETi: Bromodomain inhibitors
BRD4: Bromodomain-containing protein 4
BRD4-L: BRD4 long
BRD4-S: BRD4 short
CCNE1: Cyclin E1
DBS: Double strand breaks
DDR: DNA damage repair
ECM: Extracellular matrix
EMT: Epithelial-mesenchymal transition
EN1: Homeobox protein engrailed-1
FoxM1: Forkhead box protein M1
H3K14Ac: Histone H3 acetylate in lysine 14
H4K5Ac: Histone H4 acetylate in lysine 5
H4K8Ac: Histone H4 acetylate in lysine 8
H4K12Ac: Histone H12 acetylate in lysine 12
HAT: Histone acetyltransferase
HGSOC: High-grade serous ovarian carcinoma
HMBA: Hexamethylene bisacetamide
HMOX1: Heme oxygenase 1
HR: Homologous recombination
MCAP: Mitotic chromosome-associated protein
MEKi: MEK inhibitors
MMPs: Metalloproteinases
NHEJ: Non-homologous end joining
PARPi: Poly (ADP-ribose) polymerase inhibitors
PRG: Primary response genes
Pol II: RNA polymerase II
P-TEFb: Positive transcriptional elongation factor b
ROS: Reactive oxygen species
SE: Super-enhancers
TCGA: The cancer genome atlas
TKi: Tyrosine kinase inhibitors
TNBC: Triple negative breast cancer
YAP: Yes1 associated transcriptional regulator
